# In Vitro Study Regarding Cytotoxic and Inflammatory Response of Gingival Fibroblasts to a 3D-Printed Resin for Denture Bases

**DOI:** 10.3390/jfb16120442

**Published:** 2025-11-27

**Authors:** Miruna Dinescu, Lucian Toma Ciocan, Ana Maria Cristina Țâncu, Vlad Gabriel Vasilescu, Bianca Voicu-Balasea, Florentina Rus, Alexandra Ripszky, Silviu-Mirel Pițuru, Marina Imre

**Affiliations:** 1 Department of Prosthodontics, Faculty of Dentistry, “Carol Davila” University of Medicine and Pharmacy, 37 Dionisie Lupu Street, District 2, 020021 Bucharest, Romania; miruna.dinescu@drd.umfcd.ro (M.D.); anamaria.tancu@umfcd.ro (A.M.C.Ț.);; 2 Department of Dental Prostheses Technology, Faculty of Dentistry, “Carol Davila” University of Medicine and Pharmacy, 37 Dionisie Lupu Street, District 2, 020021 Bucharest, Romania; lucian.ciocan@umfcd.ro; 3 Department of Biochemistry, Faculty of Dental Medicine, “Carol Davila” University of Medicine and Pharmacy, 020021 Bucharest, Romania; florentina.rus-hrincu@umfcd.ro (F.R.);; 4 Discipline of Organization, Professional Legislation and Dental Office Management, Faculty of Dentistry, “Carol Davila” University of Medicine and Pharmacy, 37 Dionisie Lupu Street, District 2, 020021 Bucharest, Romania

**Keywords:** PMMA resin, 3D-printing, dentures, autophagy, biocompatibility, cytotoxicity

## Abstract

In clinical practice, the selection of dental material is a crucial factor for the final success of the treatment, regarding mechanical properties and biocompatibility. Our study aimed to evaluate the cytotoxicity of a PMMA dental resin used for denture base fabrication and to investigate whether autophagy might be involved in the response of the exposed cells. In vitro tests, such as assessments of cell viability and metabolism, nitric oxide (NO), lactate dehydrogenase (LDH), and autophagy, were conducted. The results showed that exposure to PMMA-based material decreased cell viability by 35% after 24 h and 36% after 48 h. NO levels increased by 10% after 24 h and 2% after 48 h. LDH levels increased by 8% after 24 h and 31% after 48 h. Within the limits of this present study, our results suggest a significant activation of autophagy in the exposed fibroblasts, possibly as a survival mechanism, based on the viability and cell metabolic activity results.

## 1. Introduction

A removable complete denture is a prosthetic device that replaces missing teeth and remains in contact with the oral mucosa for an extended period of time during the day [1,2]. The conventional method uses heat-polymerized acrylic resins, whereas the subtractive (milling) technique fabricates dentures from prefabricated, pre-polymerized PMMA disks, reducing residual monomer release.

Addressing the material, since the 1930s, polymethyl methacrylate (PMMA) has been the choice for the fabrication of removable dentures and continues to be the gold standard material for long-term complete dentures [3]. The influence of the polymerization cycle on the biocompatibility of PMMA resins has raised interest in the research area, demonstrating that, despite the polymerization duration, a certain amount of residual monomer is released, with methyl methacrylate (MMA) being the most common [4,5,6,7]. In contrast, acrylate resins for additive manufacturing, often called 3D-printing resins, differ significantly from conventional PMMA regarding their chemical composition and polymerization mechanism [4]. The resins used for 3D printing are predominantly based on urethane dimethacrylate (UDMA), triethylene glycol dimethacrylate (TEGDMA), or Bis-GMA oligomers [8,9].

Although several studies have examined the cytotoxicity of 3D-printed resins, most have focused on provisional restorations, splints, or aligners [5,6]. Far fewer investigations have focused on complete denture bases [10,11,12,13]. Given that edentulous patients use dentures for an extended period during the day, resulting in prolonged contact with the oral mucosa, it is clearly necessary to assess their biological behavior on human gingival fibroblasts. Moreover, most existing studies rely on evaluating cell viability, providing limited information on the potential inflammatory and autophagy responses induced by these new materials.

Therefore, regardless of the manufacturing process, all complete dentures undergo polymerization at different stages of the workflow [13]. Studies have shown that a longer polymerization cycle at higher temperatures results in lower concentrations of residual monomer, but it is not absent. It has been proven that the presence of residual monomer is a key factor in decreasing cellular viability, thereby inducing cytotoxicity [4,14]. For this reason, it is necessary to study the concentration of residual monomer that remains in the denture base and which could have consequences for biocompatibility [15].

Biocompatibility refers to the ability of an artificial material to be tolerated by the host organism without causing local or systemic adverse effects. Diminishing of this quality can lead to damage to cellular components, specifically membrane disruption, reduced cell survival rates, and ultimately, stimulation of cell death processes. Biocompatibility is most frequently assessed in vitro by evaluating the degree of cytotoxicity of acrylic resin on cell cultures. Hence, the material can be categorized into four toxicity classes: non-cytotoxic, slightly cytotoxic, moderately cytotoxic, and highly cytotoxic, the latter corresponding to a cellular non-viability rate of 75% [3].

## 2. Purpose

Our study aims to evaluate the cytotoxicity of the 3D-printed resin used for denture base fabrication and to investigate whether autophagy might play a role in the behavior of the exposed cells.

## 3. Materials and Methods

### 3.1. Specimen Preparation

Disk-shaped specimens with a diameter of 12 mm and a height of 2 mm were initially designed virtually using Meshmixer software (Meshmixer v3.5, Autodesk Inc., San Francisco, CA, USA). The STL file was then exported to a 3D printer with Digital Light Processing (DLP) technology (Creo C5, Planmeca Oy, Helsinki, Finland), setting a 90-degree printing orientation and 50 μm layer height to produce the samples using a light-curing material for 3D-printing removable denture bases (FotoDent Denture 385 nm, pink-transparent, Dreve Dentamid, Unna, Germany). The post-processing included a 10 min rinse in a 99% isopropyl bath using a Form Wash device (Formlabs, Somerville, MA, USA). After cleaning, the disks were air-dried at room temperature using a compressed air duster, followed by a 10 min polymerization cycle in a desktop ultraviolet curing chamber (Form Cure, Formlabs, Somerville, MA, USA) at 90 °C, equipped with a 405 nm LED light source with an irradiance of 14.5 mW/cm^2^.

### 3.2. Analysis of the Specimens

The elemental analysis was performed using a scanning electron microscope (SEM) (Hitachi TM3030PLUS Tabletop, Hitachi High-Tech Corporation, Tokyo, Japan) integrated with an energy-dispersive X-ray spectroscopy system (EDS, QUANTAX 70, Bruker Corporation, Billerica, MA, USA) with an integrated detector (XFlash 430H, Bruker Corporation, Billerica, MA, USA). Samples were analyzed at different magnifications: ×500, ×2000, ×5000, ×10,000, and ×25,000. These were selected to provide serial details of the sample surfaces.

### 3.3. Cell Culture

The HFIB-G cell line (Human Fibroblasts from Gingiva, Provitro, Berlin, Germany) was cultivated in Dulbecco’s Modified Eagle’s medium (DMEM, Sigma-Aldrich, Darmstadt, Germany) with 10% fetal bovine serum at a controlled temperature of 37 °C and 5% CO_2_ concentration. For all experiments, fibroblasts were placed in 24-well plates at a density of 2 × 10^4^ cells per well and allowed to attach overnight, ensuring uniform seeding in 1 mL of culture medium per well. In the next phase, the cells were incubated for 24 and 48 h with the dental material samples. The material samples consisted of disk-shaped PMMA specimens that were sterilized by immersion in 70% ethanol for 30 min, air-dried, and exposed to UV light for 30 min on each side, then placed centrally in each well to ensure consistent specimen-to-well configuration. Cells that were not exposed to dental materials were included in the control group. Incubation durations of 24 h and 48 h were selected in compliance with ISO 10993-5:2009 [16] for cytotoxicity evaluation and reflect the clinical application of PMMA dental materials, including denture bases, surgical guides, occlusal splints, orthodontic aligners, and sports mouthguards, which are removable devices and do not involve long-term contact.

### 3.4. Cell Viability Assay

3-(4,5-dimethylthiazol-2-yl)-2,5-diphenyltetrazolium bromide (Sigma-Aldrich, Darmstadt, Germany) was applied to assess cell proliferation and cytotoxicity via a colorimetric approach, which offers high sensitivity and produces a signal that is directly proportional to the number of viable cells. The mechanism of this test is based on the enzymatic reduction of yellow tetrazolium salt (MTT) to insoluble purple formazan crystals by metabolically active cells. Following formazan formation, the crystals were solubilized, and the absorbance was measured at 595 nm to determine the relative cell viability. Thus, a working solution of MTT at 1 mg/mL was prepared. The cells were treated with 200 µL of cell culture medium and 200 µL of MTT solution for 4 h. Subsequently, 400 µL of isopropanol was added to dissolve the resulting formazan crystals. FLUOstar^®^ Omega multi-mode microplate reader (BMG LABTECH, Ortenberg, Germany) was used to measure absorbance at 595 nm.

### 3.5. Griess Assay

Nitric oxide (NO) production was quantified by measuring the nitrite levels in the cell culture supernatants using the Nitric Oxide Assay Kit (Thermo Fisher Scientific, Vienna, Austria, Catalog number: EMSNO). Culture media samples were collected after 24 h and 48 h of incubation to assess changes in NO levels, which are known to increase during inflammation and apoptosis triggered by cytotoxic effects. The collected medium was mixed with the kit’s reagents (Griess Reagent I and Griess Reagent II, 1:1). After 10 min of reaction, the absorbance was measured spectrophotometrically. Optical density readings were obtained at 540 nm using FLUOstar^®^ Omega (BMG LABTECH, Ortenberg, Germany).

### 3.6. Lactate Dehydrogenase (LDH) Assay

Cell membrane integrity was assessed by measuring extracellular LDH release in culture media collected after 24 h and 48 h of exposure to dental material PMMA-based. LDH activity was determined using commercial LDH Cytotoxicity Assay kit from Thermo Fisher Scientific (Eugene, OR, USA, REF: C20300), according to the manufacturer’s instructions. Therefore, 50 μL of the sample was combined with 50 μL of a reaction mixture containing dye, substrate, and cofactor. After a 30 min incubation in the dark, the absorbance was measured at 490 and 680 nm using FLUOstar^®^ Omega (BMG LABTECH, Ortenberg, Germany).

### 3.7. Fluorescence Staining Assays

For autophagy assessment, the CYTO-ID^®^ Autophagy Detection Kit (catalog number: ENZ-51031, Enzo Life Sciences, Inc., Farmingdale, NY, USA) was used after exposing fibroblasts to dental material samples for 48 h, according to the manufacturer’s protocol. In brief, the gingival fibroblasts were incubated for 48 h at 37 °C and 5% CO_2_ in the presence of the samples. After the incubation time, the cells were washed with 1X Assay Buffer and kept for 30 min at 37 °C, protected from light, in the presence of Microscopy Dual Detection Reagent. Images were captured using an inverted fluorescence microscope (IM-3LD4D, OPTIKA S.R.L., Ponteranica, Italy), and the evaluation was performed using the ImageJ software (version 1.54a). Subsequently, the quantification results were compared with those of the control group.

### 3.8. Statistical Analysis

The data obtained from the test and control samples were statistically analyzed using Microsoft Office Excel (mean, ratio, standard deviation, and *t*-test function). The *p*-values ≤ 0.05 were considered statistically significant. The one-way ANOVA revealed a significant overall effect of group on the measured outcome, F(5, 30) = 103.30, *p* < 0.001, indicating substantial differences across the six conditions.

## 4. Results

### 4.1. Scanning Electron Microscope (SEM) Analysis

**Figure 1:**
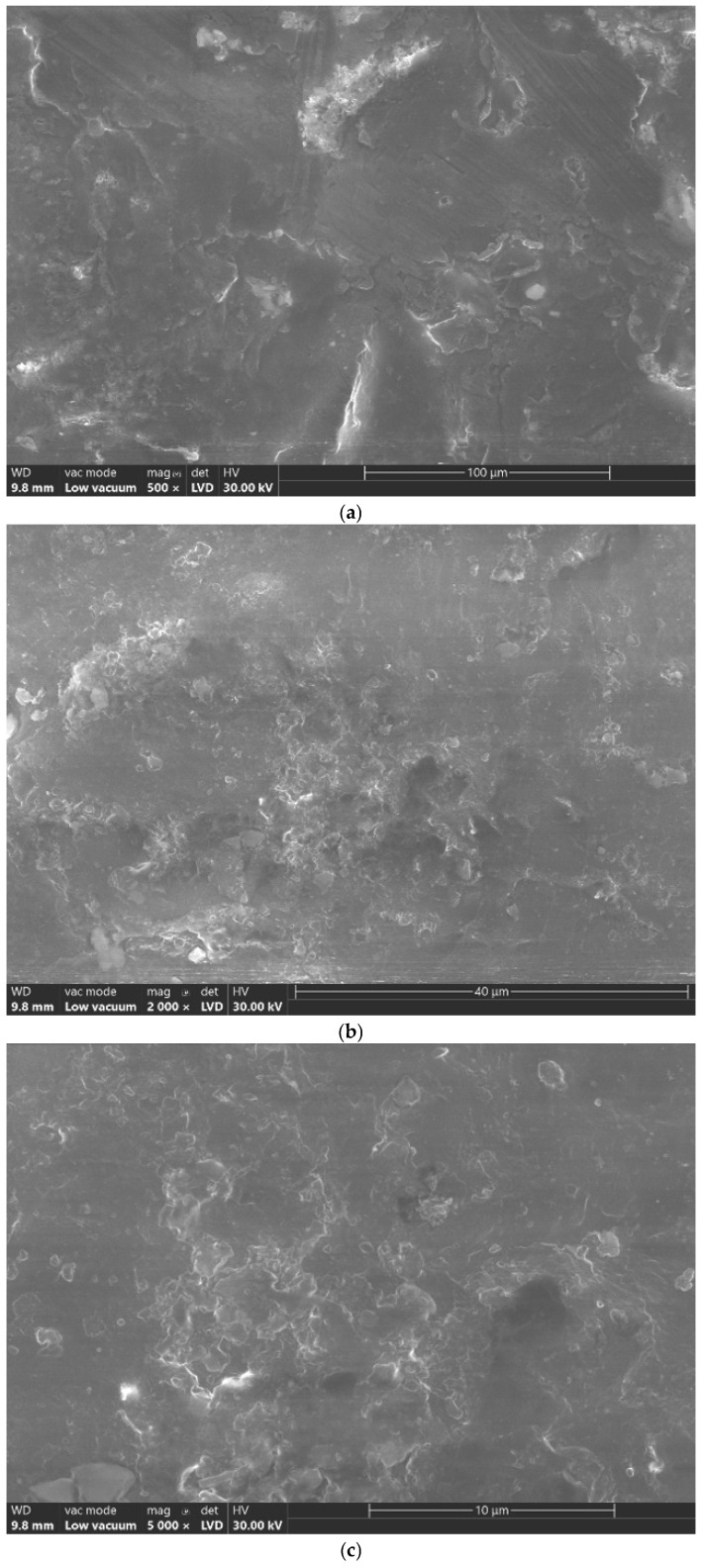

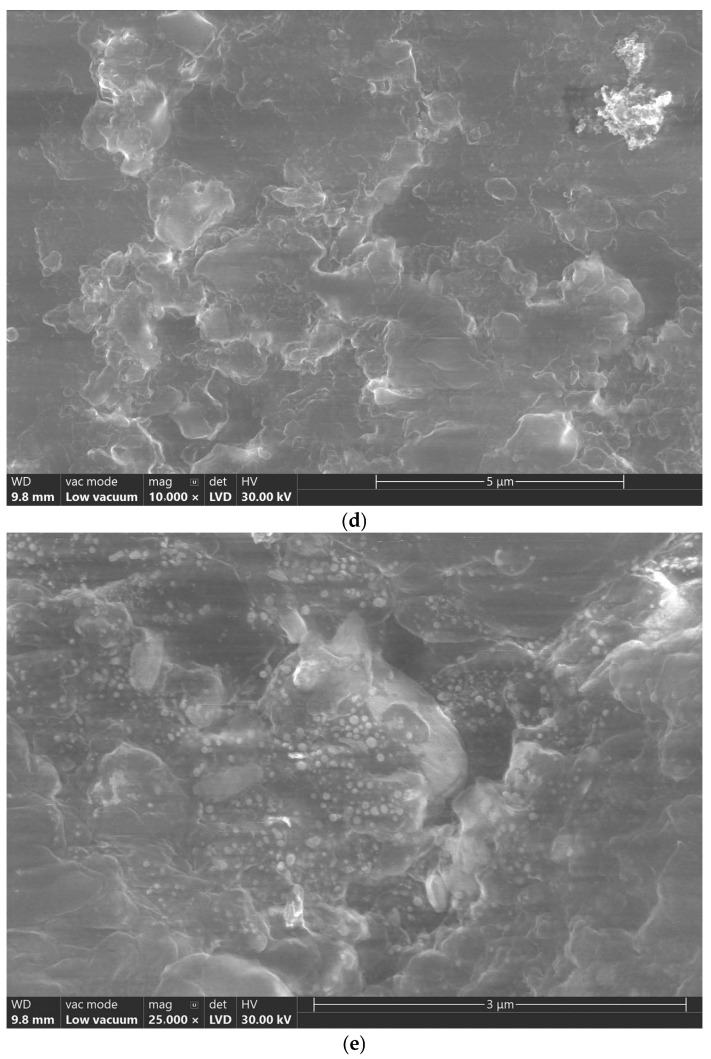
SEM imaging of 3D-printed denture base PMMA surfaces. (**a**) Surface morphology (magnification 500×); (**b**) Surface morphology (magnification 2000×); (**c**) Surface morphology (magnification 5000×); (**d**) Surface morphology (magnification 10,000×); (**e**) Surface morphology (magnification 25,000×).

The SEM analysis (Figure 1) showed a lack of homogeneity of the material surface at different magnifications. The interlocking polymer matrices are irregular, presenting disruptions on the material surface. At high resolution magnification (25,000×), it can be noticed that particles of not chemically reacted monomer are embedded into the surface of the polymeric matrix.

### 4.2. Cell Viability Analysis

**Figure 2:**
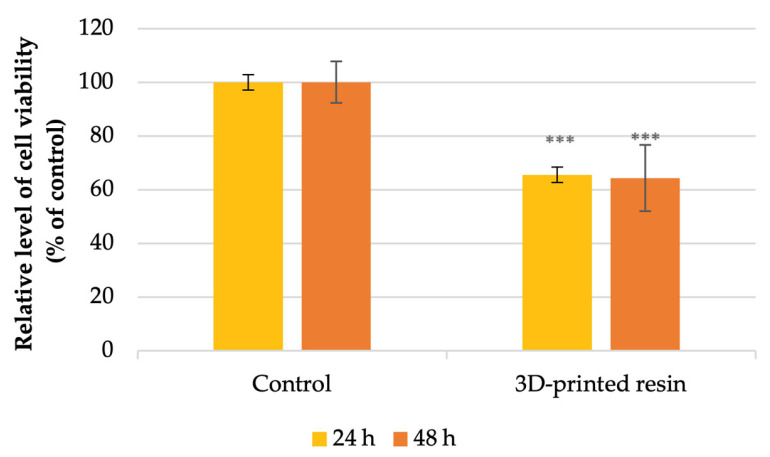
Impact of the 3D-printed denture base PMMA on HFIB-G cells viability quantified after 24- and 48 h of incubation, compared to control (unexposed cells). Results are means ± SD (n = 6). *** *p* < 0.001.

The MTT assay showed that the tested material decreased cell viability after 24 h and 48 h of incubation compared to the control (Figure 2). After 24 h of incubation, the 3D-printed material decreased the viability of human gingival fibroblasts with 35% of the control level. After 48 h of exposure to the PMMA material, there was no significant difference, and the MTT level was 36% lower than the control level.

**Figure 3:**
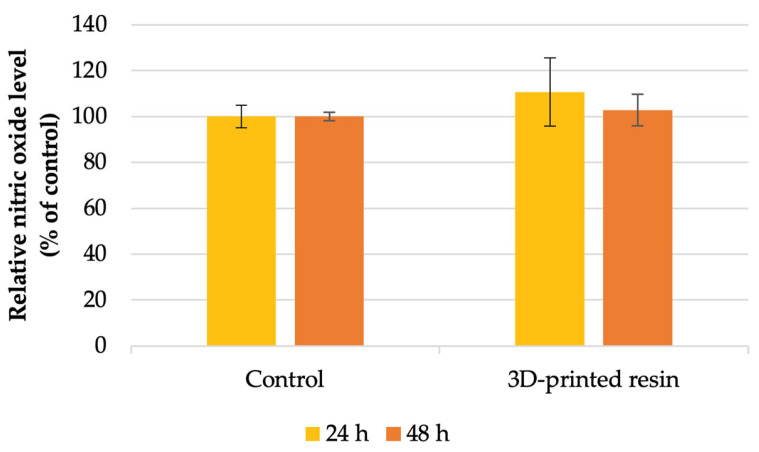
Nitric oxide level measured after the 24 h and 48 h incubation of HFIB-G cell line for the 3D-printed denture base PMMA, compared to control (unexposed cells). Results are means ± SD (n = 6).

Following cell viability assessment, the study further examined the inflammatory response by measuring nitric oxide levels. NO levels increased after exposure to the tested material for both incubation periods compared to the control (Figure 3). The highest increase was observed after 24 h of incubation, where the level was 10% greater than that of the control. However, after 48 h of incubation, the results revealed a slight increase in NO levels compared to the control. Therefore, the results were not statistically significant.

**Figure 4:**
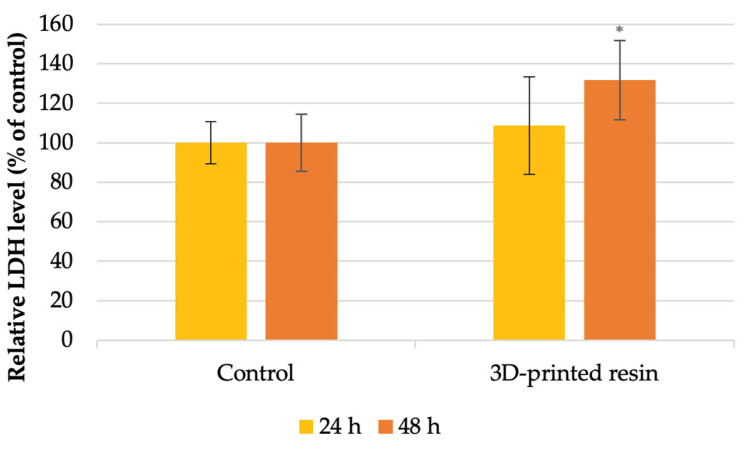
Lactate dehydrogenase (LDH) levels released after the 24 h and 48 h incubation of HFIB-G cells with the 3D-printed denture base PMMA, compared to control (unexposed cells). Results are means ± SD (n = 6). * *p* < 0.05.

Regarding the level of lactate dehydrogenase (Figure 4), the highest increase, 32%, was observed after 48 h of incubation with PMMA, which was in concordance with the decrease in cell viability. After 24 h, a slight increase of approximately 8% was observed compared to the control.

### 4.3. Autophagy Assessment

**Figure 5:**
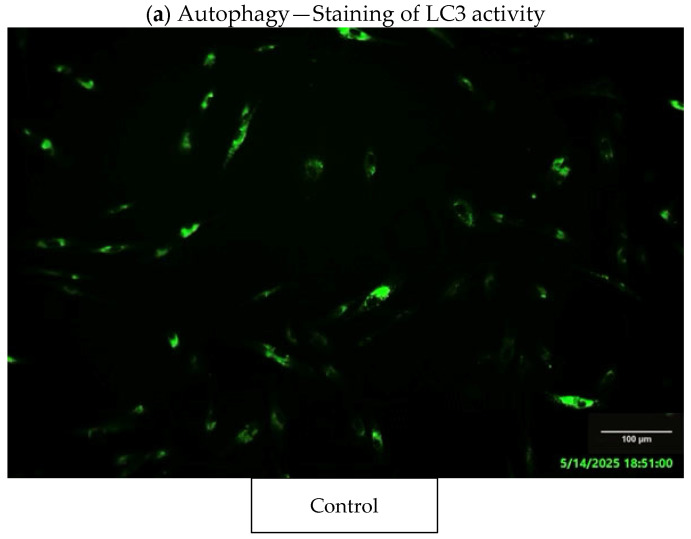

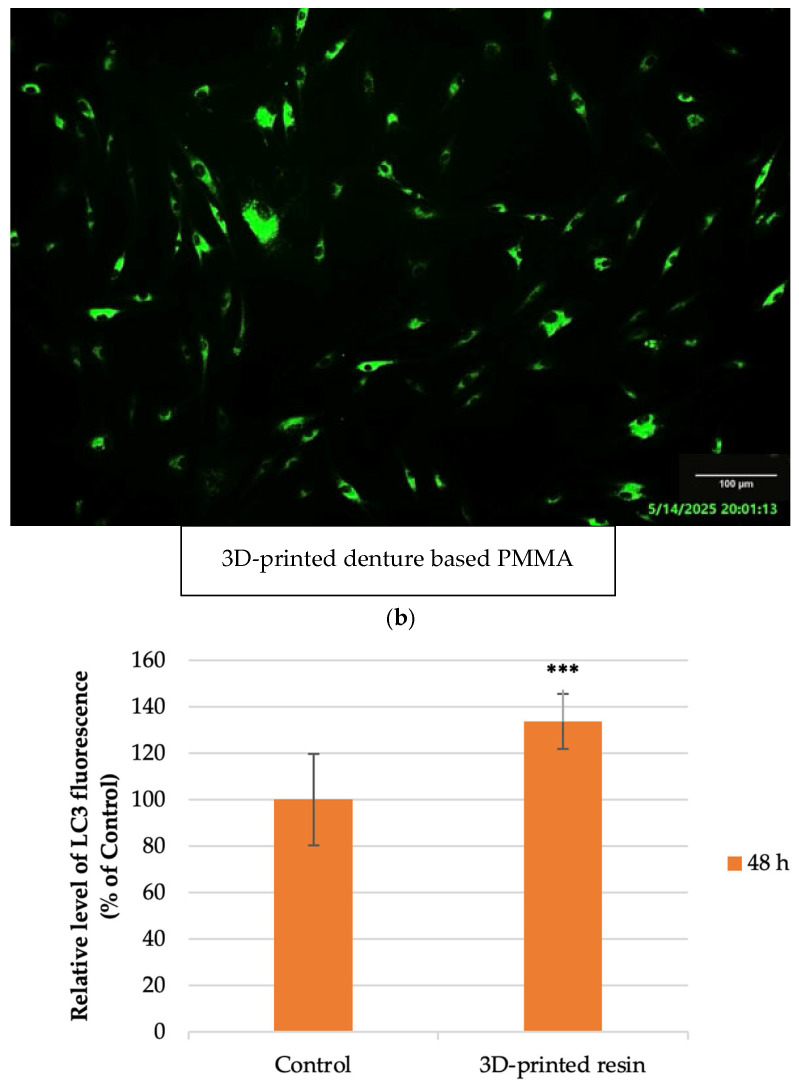
Activation of autophagy after 48 h of exposure of gingival fibroblasts to the 3D-printed material. The control is represented by unexposed cells. (**a**) Representative images of fluorescent labeling of the autophagosome marker LC3. Scale bar: 100 µm; (**b**) Quantification of the fluorescence intensity of the LC3 protein. Results are calculated as mean ± SD (n = 9) and reported to the control. *** *p* < 0.001 compared to the control.

After 48 h exposure of human gingival fibroblasts to 3D-printed denture base PMMA, staining for LC3 protein activity indicated a higher fluorescence intensity compared to the control (un-exposed cells) (Figure 5a), with a 33% increase over control in LC3 fluorescence intensity (Figure 5b), suggesting a more pronounced activation of autophagy after incubation with the dental material. In addition, a pronounced perinuclear distribution of autophagosomes was observed in the case of exposure to the dental material compared to the control.

## 5. Discussion

Computer-aided design and manufacturing (CAD-CAM) continues to evolve and transform the workflow of complete denture fabrication from analog to digital. Whether it is subtractive milling or additive manufacturing, like Three-dimensional printing, it represents an alternative to traditional methods [17,18,19]. 3D printing, especially digital light processing (DLP), offers advantages such as short production times, minimal waste material, and accurate reproduction of anatomical morphologies, including undercuts or complex areas on the intaglio side, characteristics that are often limited in reproducing with milled dentures due to the diameter and accessibility of the burs [8,20,21,22,23]. However, there are still several drawbacks that persist. The post-processing step is critical because an incomplete phase of polymerization can lead to dimensional modifications, residual monomers, or surface roughness, all of which weaken the fit of the denture and the biocompatibility [6,7].

Regarding the workflow of manufacturing 3D-printed dentures, the first step is the impression, which can be either analog, a conventional impression, or digital using an intraoral scanner. Although the intraoral scanners industry is improving the process, capturing soft tissue, border extensions, and registering jaw movement in fully edentulous patients remains challenging [22,24]. Therefore, conventional impressions are still preferred by clinicians, as they are essential for obtaining a good peripheral seal and retention. The intaglio surface of the denture is very important. It is known that poor adaptation to the mucosa may induce tissue aggressions, such as ulcerations, soreness, pain, inflammation, and progressive bone resorption, due to incorrect force distribution during the mastication process. Poor surface finishing may also produce frictional irritation and mucosal inflammation [23,24,25,26].

Although 3D printing offers excellent accuracy and customization for dentures, clinical success depends on an accurate registration of the entire edentulous ridge and a correct occlusal recording [27]. Therefore, without being attentive from the beginning steps of denture fabrication, it can lead to issues such as poor fit, tissue inflammation, and unstable occlusion. If problems arise, the properties of 3D-printed resins might even worsen tissue damage and inflammation [21,23,24].

Besides following the appropriate protocol for data acquisition, the selection of the material is also important. It requires a deep understanding of polymer chemistry, curing, and post-processing protocols to ensure that digitally fabricated dentures meet the same standards of reliability and biocompatibility as those established by conventional methods [28].

Heat-polymerized resins are composed predominantly of pre-polymerized poly(methyl methacrylate) (PMMA) powder and methyl methacrylate (MMA) monomer. During mixing and heat activation, the MMA monomer polymerizes within the PMMA matrix, forming a homogeneous acrylic structure with a high degree of conversion and a reduced amount of unreacted MMA monomer [6,7,19].

Referring to our testing material, according to the manufacturer’s official Safety Data Sheet (SDS) [29], the 3D-printed light-curing resin is composed mainly of methacrylate oligomers and a phosphine oxide photoinitiator system. Its key components include:

- Poly[oxy(methyl-1,2-ethanediyl)], α,α’-(2,2-dimethyl-1,3-propanediyl)bis[ω-[(1-oxo-2-propenyl)oxy]-] (25–50%)—a difunctional polyether dimethacrylate that provides flexibility and reduces viscosity.
- 7,7,9(7,9,9)-trimethyl-4,13-dioxo-3,14-dioxa-5,12-diazahexadecane-1,16-diylbismethacrylate (25–50%)—a urethane dimethacrylate (UDMA) derivative that forms the structural backbone, enhancing hardness and toughness.
- Aliphatic urethane triacrylate (10–25%)—a highly cross-linkable oligomer improving rigidity, scratch resistance, and surface hardness.
- Diphenyl(2,4,6-trimethylbenzoyl)phosphine oxide (TPO) (1–3%)—a photo-initiator that generates free radicals upon exposure to visible or UV light (≈405 nm), initiating polymerization.

In this case, the polymerization occurs via photo-initiated radical reactions induced by UV or visible light, resulting in a cross-linked but often less homogeneous polymer network than conventional heat-polymerized resin, with unreacted dimethacrylates such as UDMA- and TEGDMA-derived oligomers. Understanding this distinction is essential for choosing the right indications for use and assessing biocompatibility [8,9].

The SDS classifies our tested material as Skin Sensitization Category 1A, consistent with the known irritant potential of methacrylate monomers. Even if it contains no substances with recognized carcinogenicity or mutagenicity, diphenyl(2,4,6-trimethylbenzoyl)phosphine oxide is listed as a reproductive Toxicant Category 2 (H361f: suspected of damaging fertility) [29]. However, these risks are associated with an incomplete polymerization process; once polymerization and post-curing are fully achieved, it significantly reduces their release and cytotoxicity, and classifies them as non-cytotoxic or slightly cytotoxic, comparable to conventional resins [6,28,30].

Several studies have reported that conventional heat-polymerized PMMA used for denture base manufacturing induces only minimal cytotoxic effects in vitro, with fibroblast viability typically remaining above 85–100% after 24 h of exposure, and therefore being classified as non-cytotoxic [31,32,33,34]. On the other side, autopolymerized chairside materials, which possess substantially higher residual monomer content, consistently demonstrate greater cytotoxicity than heat-polymerized PMMA [35].

CAD/CAM-milled PMMA disks, due to their high degree of pre-polymerization, generally demonstrate equal or slightly better biocompatibility than conventional heat-cured PMMA, with most studies reporting fibroblast viability values above 75–80%, and superior biocompatibility compared with 3D-printed resins, due to their highly cross-linked, low-solubility structure [36]. By comparison, 3D-printed methacrylate resins used for splints or denture applications have indicated a tendency toward higher residual monomer release and more marked reductions in cell viability, particularly when post-curing is incomplete or surfaces are left unpolished, causing inflammation [6,30,37]. And when tissue health is already compromised, the chemical and mechanical properties of printed resins can worsen these issues, prolonging healing and reducing comfort [22,30]. While some photoinitiated denture base resins show minimal cytotoxicity under optimized polymerization conditions [38], others, such as polyurethane-based resins, exhibit accentuated reduced cell viability and even toxic responses [39]. In our study, the resulting ≈65% fibroblast viability still falls below the values typically reported for heat-cured or milled PMMA. Therefore, these comparisons indicate that our tests are consistent with the literature.

Consequently, our study focused on human gingival fibroblasts because the fibroblast-rich gingival tissue often comes into contact with or is near cytotoxic polymer-based dental restoration materials. Oral fibroblasts, especially human gingival fibroblasts (HGFs), are essential for evaluating the biocompatibility of dental materials. Fibroblasts actively participate in cellular immune responses in connective tissues when activated by signals such as cytokines and bacterial products, which are abundant at inflammatory sites that may develop at the border between dental material and gingiva. Furthermore, fibroblasts can also produce proinflammatory cytokines and present antigens to proliferating T cells [40]. The specific cytokines produced by immune-activated fibroblasts depend on the stimulus and are regulated at multiple levels, including transcription, translation, and posttranslational modifications. Generally, cytokines that have the potential to cause tissue damage, such as IL-1α and, to a lesser extent, IL-6, are more tightly controlled than cytokines with more restricted target cell specificity, like CSFs [40,41]. The mechanisms behind cytokine production by fibroblasts have important implications for both normal connective tissue homeostasis and pathological conditions.

Autophagy involves transporting various substances, such as proteins and organelles, to the lysosome for degradation and recirculation, thereby supporting normal cellular functions. Recent research highlights autophagy’s role in influencing inflammatory responses, especially in cytokine production. Different proinflammatory cytokines can regulate autophagy induction: IL-1, TNF-α, and IL-17 promote it, while IL-13, IL-33, IL-10, and IL-14 inhibit it. Moreover, autophagy activation can either enhance or suppress cytokine production and secretion, impacting inflammation regulation. Since cytokine levels regulated by autophagy significantly affect the pathophysiology of inflammation-related diseases, understanding the link between autophagic flux and pro-inflammatory mediators is crucial [42,43].

Our MTT tests assessing cell viability and metabolism indicated that the dental materials tested reduced cell viability after 24 and 48 h of incubation compared to the control group (Figure 1). Specifically, after 24 h, the tested 3D-printed resin lowered the viability of human gingival fibroblasts to 35% of the control. After 48 h of exposure to the PMMA material, there was no significant difference, with the MTT level being 36% lower than the control. According to ISO 10993-5:2009 standards [16] for in vitro cytotoxicity, a material is considered cytotoxic if it decreases cellular viability by more than 30% relative to untreated cells. Furthermore, the level of LDH (Figure 3) showed that the highest increase was observed after 48 h of incubation with PMMA, which was in concordance with the decrease in cell viability. After 24 h, a slight increase of approximately 8% was observed compared to the control. Regarding the nitric oxide (NO) levels, it was observed that levels increased following exposure to the tested material during both incubation periods compared to the control; however, these increases were not statistically significant. Autophagy, a process of self-digestion, is a vital cellular mechanism in eukaryotic cells that controls growth, cell death, and energy management. It plays a critical role in removing damaged organelles, misfolded or aggregated proteins, and pathogens. Damaged components are enclosed within a double-membrane vesicle called an autophagosome, which transports them to lysosomal enzymes for breakdown. Afterwards, the contents are released into the cytoplasm for biosynthesis and energy generation. Therefore, autophagy is essential for preserving cellular homeostasis and reacts to stimuli like nutrient deprivation, oxidative stress, and contact with dental materials to prevent cell damage [43,44,45]. The lipidated form of microtubule-associated protein light chain 3 (LC3), known as LC3-II, serves as an autophagosome marker; its formation and turnover are indicators of autophagy activity [45,46]. Our results showed a significantly higher LC3 fluorescence level in the exposed fibroblasts, indicating increased autophagic activity, possibly as a survival mechanism, based on the viability and cell metabolic activity results. Autophagy can serve both as a survival mechanism and a pathway to cell death. Normally, basal autophagy helps maintain cellular balance by degrading excess or unnecessary components. During stress, such as contact with dental material surfaces, autophagy may increase to support cell survival by breaking down proteins and organelles. However, prolonged and intense stress can cause overactivation of autophagy, leading to excessive degradation of cytoplasmic proteins and organelles [43,47]. At this point, autophagy shifts from being protective to destructive, resulting in autophagy-driven cell death. Its role in cell fate is especially notable in cancer, where it can both suppress and promote tumor progression [48,49]. Consequently, controlling autophagy should become one of the major focuses in dental materials’ biocompatibility studies. Recent studies have also shown that autophagy is linked to proinflammatory cytokines, increasing TNF-α and IL-8 but decreasing IL-1α, IL-1β, and IL-18 in macrophages and dendritic cells [50]. Nevertheless, how autophagy influences the molecular mechanisms behind the biocompatibility of dental materials is still not well understood. Autophagy is the main catabolic process contributing to cell survival under stress conditions and other intrinsic and extrinsic insults, such as the dental material [48]. However, the association of autophagy with the adverse effects of dental PMMA-based dental materials has not been extensively investigated, although autophagy is known to be involved in various types of inflammation [50,51]. Our results are consistent with the literature reports [52,53].

Furthermore, the quick activation of autophagy largely relies on protein post-translational modifications (PTMs), which serve as molecular switches that initiate signaling cascades. Disruptions in autophagy are linked to pathological states, including inflammatory reactions that sometimes occur at the contact point between dental materials and the gingiva.

Indeed, our results also revealed that exposure to the oligomer-based material induced a significant increase in autophagy levels, given that the NO level in the exposed cells was not significantly different from the control.

Despite its strengths, this study has some limitations that should be considered. Most importantly, the biocompatibility assessment was performed using only one type of 3D-printed dental resin and a single 3D printer. Future research should include multiple brands and formulations to provide a more comprehensive understanding of material variability and performance. Because the chemical composition of resins, polymerization time, and post-processing procedures can differ significantly among manufacturers and technologies, the results cannot be generalized to all printable denture base materials. Another limitation could be the exposure time, which is limited to 24 and 48 h, without studying the possible effects of the prolonged term, when residual monomer could still be released. In addition, this study did not quantify residual monomer release, which limits the ability to directly correlate the observed cytotoxicity with the material’s chemical composition. Further research should include chemical elution analysis to clarify this relationship. Although we reviewed and used validated methods for the cytotoxicity assessment, our evaluation was limited to in vitro conditions, which may not sufficiently demonstrate the complex in vivo oral cavity conditions. The lack of long-term clinical data also limits our ability to draw final conclusions about the biological safety of newer 3D-printed resins, but we consider that future in vivo studies are essential to confirm the clinical biocompatibility of these materials.

## 6. Conclusions

This study evaluated the cytotoxic and stress-related cellular responses of a 3D-printed methacrylate-based denture base resin using human gingival fibroblasts. The analysis included cell viability, nitric oxide (NO), lactate dehydrogenase (LDH) and autophagy-related LC3 expression.

The main findings were:

- Cell viability: decreased by 35% at 24 h and 36% at 48 h.
- Nitric oxide production: increased by 10% after 24 h and 2% after 48 h.
- LDH release: increased by 8% at 24 h and 31% at 48 h, indicating membrane damage.
- Autophagy marker (LC3): significantly elevated fluorescence levels in exposed fibroblasts.

Further in vivo and long-term studies are needed to determine the clinical relevance of these responses and to support comprehensive biocompatibility evaluation of additively manufactured denture base materials.

## Data Availability

The original contributions presented in the study are included in the article; further inquiries can be directed to the corresponding authors.

## Abbreviations

The following abbreviations are used in this manuscript:

3D-printing	Three-dimensional printing
Bis-GMA	Bisphenol A-glycidyl methacrylate
CAD-CAM	Computer-aided design and computer-aided manufacturing
CSF	Cytokines in the cerebrospinal fluid
DLP	Digital light processing
DMEM	Dulbecco’s Modified Eagle Medium
HFIB-G	Human fibroblast–gingiva cell line
HGF	Human gingival fibroblasts
IL-1	Interleukin-1
IL-10	Interleukin-10
IL-13	Interleukin-13
IL-14	Interleukin-14
IL-17	Interleukin-17
IL-18	Interleukin-18
IL-1α	Interleukin-1 alpha
IL-1β	Interleukin-1 beta
IL-33	Interleukin-33
IL-6	Interleukin-6
IL-8	Interleukin-8
LC3	Microtubule-associated protein light chain 3
LC3-II	Microtubule-associated protein light chain 3
LDH	Lactate dehydrogenase
MTT	3-(4,5-Dimethylthiazol-2-yl)-2,5-diphenyltetrazolium bromide
NO	Nitric oxide
PMMA	Polymethyl methacrylate
PTMs	Protein post-translational modifications
SDS	Safety Data Sheet
SEM	Scanning electron microscopy
STL	Standard tessellation language
T cells	Thymus-derived lymphocytes
TEGDMA	Triethylene glycol dimethacrylate
TNF-α	Tumor necrosis factor alpha
TPO	Diphenyl(2,4,6-trimethylbenzoyl)phosphine oxide
UDMA	Urethane dimethacrylate
UV	Ultraviolet
